# Laryngeal Preservation in Managing Advanced Tracheal Adenoid Cystic Carcinoma

**DOI:** 10.1155/2015/404586

**Published:** 2015-03-24

**Authors:** Thavakumar Subramaniam, Paul Lennon, John Kinsella, James Paul O'Neill

**Affiliations:** ^1^Department of Otolaryngology, Head and Neck Surgery, St James Hospital, Dublin, Ireland; ^2^Department of Otolaryngology, Head and Neck Surgery, Beaumont Hospital, Dublin, Ireland

## Abstract

A 37-year-old male athlete was diagnosed with primary tracheal adenoid cystic carcinoma following investigation for dyspnea, wheeze, and eventual stridor. Preoperative bronchoscopy revealed a highly vascular tumor 4 cm distal to the cricoid with no gross disease extending to the carina. Imaging revealed circumferential tracheal irregularity immediately inferior to the cricoid, with no definite cricoid invasion. Locoregional extension of disease was noted invading the thyroid and abutment of the carotid approximately 180°. Intraoperative findings identified tracheal mucosal disease extending distal to the carina and proximally at the cricothyroid joints where bilateral functional recurrent nerves were preserved. A decision made to preserve the larynx given the inability to fully resect distal tracheal disease. A 5 cm sleeve resection of the trachea was made with a cricotracheal anastomosis following suprahyoidal muscle release and laryngeal drop-down. The patient was treated with adjuvant radiotherapy including platinum based chemotherapy in an effort to maximise local control. PET scanning three months after therapy revealed no FDG uptake locally or distally.

## 1. Introduction

Adenoid cystic carcinoma is the most common tumour of minor salivary glands; however primary tracheal adenoid cystic carcinoma is a rare occurrence. The classic management involves surgical resection and adjuvant therapy; however achieving clear surgical margins in this disease and the implication of distant dissemination make for the challenging oncological management [1, 2].

## 2. Case Report

**Figure 1:**
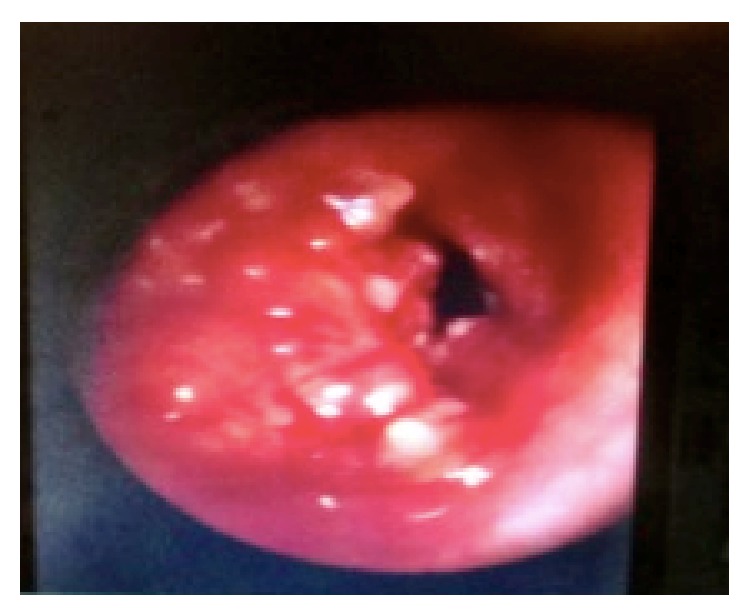
Rigid bronchoscopy finding of a firm well-defined mass with significant tracheal obstruction.

We report a case of a 37-year-old professional male athlete presenting with a seven-month history of worsening respiratory function. He was diagnosed with asthma and managed in the community. He was referred for further investigation following worsening of his symptoms and the onset of stridor. Flexible nasendoscopy revealed intact vocal cord function with a mass lesion visible in the trachea. Rigid bronchoscopy reported an intraluminal tracheal mass immediately inferior to the cricoid extending five centimeters caudally resulting in eighty percent tracheal obstruction (Figure 1). The carina and upper oesophagus were noted to be grossly free of disease. A biopsy of the mass diagnosed tracheal adenoid cystic carcinoma of cribriform and tubular variant.

MRI scan and PET-CT demonstrated a low to intermediate FDG uptake of 5.9. Key findings from imaging included submucosal extension within the tracheal lumen, invasion of thyroid gland, and no direct invasion of the cricoid cartilage. There was no evidence of cervical nodal enhancement or distant dissemination. The patient's case was discussed at our multidisciplinary team meeting with input from cardiothoracic surgery, medical oncology, radiation oncology, and the head and neck team. Tracheal resection with primary cricotracheal anastomosis, total thyroidectomy, and preservation of the larynx was the proposed surgical intervention.

**Figure 2:**
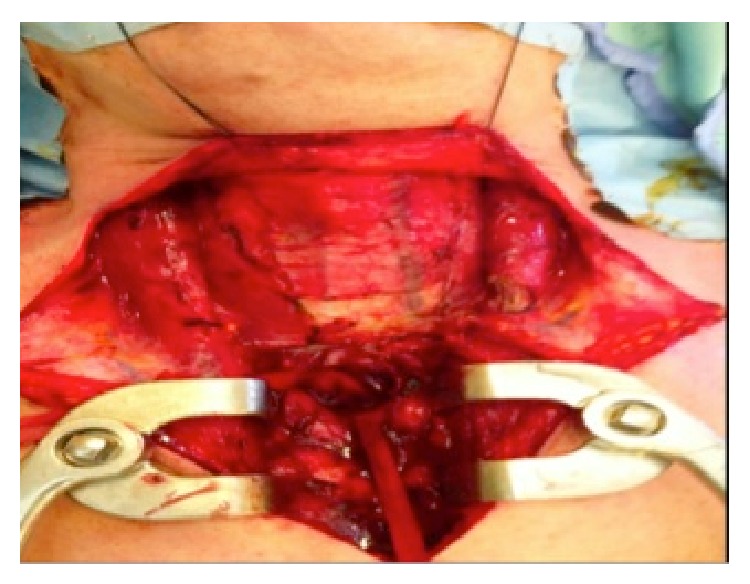
Surgical approach with T incision and sternal split providing adequate access.

T-shaped neck incision at the level of cricoid cartilage was made with a partial sternotomy to afford greater access (Figure 2). Vessel loops were applied to the innominate artery and vein. Mobilization of the thyroid lobes was then performed with identification and preservation of the recurrent laryngeal nerves bilaterally. A small pocket of disease was identified bilaterally at the cricothyroid joints surrounding both recurrent laryngeal nerves. Inspection was then made of the distal aspect of the dissection where submucosal extension of disease in the trachea was also identified tracking caudad towards the carina. A curative resection was clearly not a possibility. A 5 cm resection of the trachea was performed to achieve gross intratracheal disease clearance. Following the tracheal resection a cricotracheal anastomosis was performed, using 3-0 polydioxanone sutures preserving the recurrent nerves at the cricothyroid junction. The tension on the cricotracheal anastomosis was reduced by suprahyoidal muscle release and its strength augmented using Tisseal on the anastomotic line. Postoperative care included suturing of the patients chin to his chest with 0-nylon sutures, ensuring the patient's head was kept flexed and thus reducing tension on the new anastomosis. An alternative to this method is with the use of a custom made neck brace. The patient was sedated and intubated in intensive care unit for 7 days prior to extubation in operating theatre. Mild surgical emphysema was noted but resolved by day 3 after operation. The patient was also treated with broad-spectrum antibiotics, a tapering dose of corticosteroids and continuous humidified oxygen and saline nebulisers.

Chin to chest sutures were removed at day 14 after operation and patient was discharged home. Three weeks post-op the patient maintained normal voice quality, normal deglutition and showed significant improvement in exercise tolerance.

**Figure 3:**
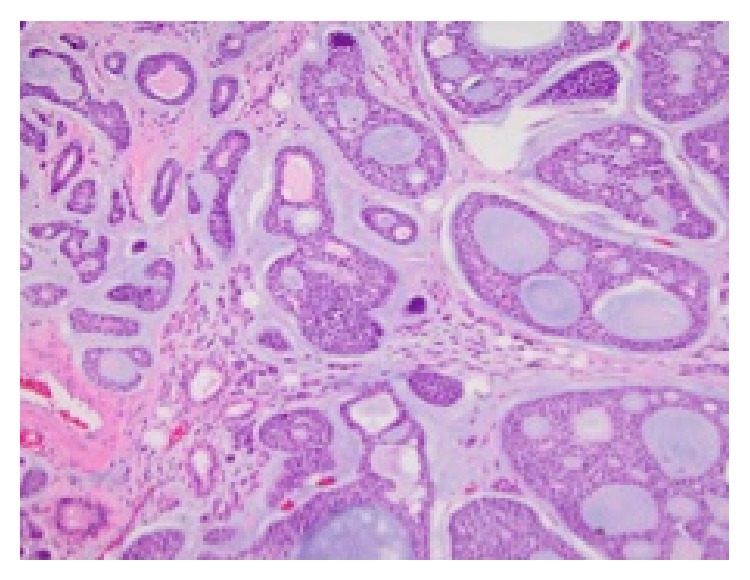
Biphasic ducts and basal myoepithelial cells with cystic spaces.

Histopathological analysis of the operative specimen confirmed adenoid cystic carcinoma with a predominately cribriform pattern (Figure 3). The disease involved multiple tracheal rings with extensive perineural and lymphovascular invasion. The tumor invaded the thyroid gland and both the superior and inferior margins were positive for tumor involvement.

Following discussion at our MDT meeting a decision was made to give adjuvant radiation and concurrent platinum based chemotherapy given the potential locoregional advantage its inclusion may afford [3]. A total radiation dose of 60 Gy and a cisplatin dose of 30 mg/m^2^ were administered under the care of the medical and radiation oncology team. No severe or serious adverse events were reported.

A follow-up PET scan performed 3 months after adjuvant radiation and chemotherapy showed no FDG uptake both locoregionally and distally. The patient has also returned to playing football at professional level maintaining an excellent level of pulmonary function, phonation, and deglutition.

## 3. Discussion

Primary tracheal malignancy is a rare occurrence accounting for 0.2% of all respiratory malignancies in the United States [4] or an annual incidence of approximately 1 per 1 million [4, 5]. The two most common histological types are squamous cell carcinoma (SCC) and adenoid cystic carcinoma (AdCC), accounting for 49% and 23% of 1404 tracheal malignancies, respectively [5]. Diagnosis is often delayed, as symptoms are subtle and insidious and often misdiagnosed as asthma, as in our case [6]. Tumors may not cause symptoms until they occlude 75% of the tracheal luminal diameter [6, 7].

Surgical excision remains the mainstay of treatment for primary adenoid cystic carcinomas of the trachea. The trachea is a fibroelastic tube supported by incomplete cartilaginous rings. The adult trachea has a mean length of 11 cm (cricoid to carina) with a range of 9–15 cm. The amount of trachea that can safely be excised varies substantially between patients. Young age, thin body habitus, and long neck are favorable patient factors for long tumors. Tracheal mobilization manoeuvres include extreme flexion of the neck (1 to 6 cm), incising the annular ligaments between tracheal rings (1 to 2 cm), suprahyoid or infrahyoid release of the upper laryngotracheal unit (2.5 to 5 cm), and blunt dissection and mobilization of the lower tracheal segment (0.5 to 1 cm). A combination of laryngeal release procedures, blunt mobilization of the lower tracheal segment, and neck flexion will yield about 4 to 6 cm of mobilization depending upon the patient's age and range of neck motion [8]. Further care must be made to prevent vascular disruption. Five to six centimetres is usually the maximal length that can be safely resected with a primary anastomosis [9]. Postoperatively the neck is maintained in extreme flexion with use of either a halo splint or heavy nylon suture between skin and soft tissues of the submental region and the skin and soft tissues of the sternal region for 2-3 weeks [10]. Subcutaneous emphysema is the most common complication [10]. Wurtz et al. [11] describe a novel surgical technique using an aortic allograft to enable extended tracheal resection. The authors report an 83% complete resection (*R*0) rate from a cohort of 6 patients with tracheal minor salivary gland malignancy. An aortic allograft is interpositioned following tracheal resection splinted by a silicon stent. A pectoral muscle flap is then wrapped around the graft to promote revascularization and prevent fistula formation.

Many reports recommend adjuvant radiotherapy (RT) for all patients with adenoid cystic carcinomas to improve local control rates and improve disease free survival [12, 13]. Radiation alone has poorer outcomes; however this in part may be due to some selection bias, with more advanced tumours likely to be deemed unresectable [6, 9]. For unresectable cases proton irradiation [12] or high linear energy transfer with neutron or carbon ions has also shown promise as higher radiobiological effectiveness and added precision allow for higher dose intensity [13]. These treatments are however limited by cost and availability.

Combined chemoradiation is however widely available. There is some evidence that concurrent chemoradiotherapy, especially with platinum based chemotherapy, may have a role in improving locoregional control [3, 14–16]. In one study 13 of 16 patients with unresectable disease were still alive with a median follow-up of 25 months [3]. Of particular interest is a recent case report of an extensive unresectable tracheal AdCC that has been successfully treated with chemoradiotherapy [17].

Allen et al. [17] reported the use of radiation and concurrent carboplatin and paclitaxel for a case of unresectable tracheal adenoid cystic carcinoma. The authors report on success with this treatment regime as surveillance biopsies in this case showed no disease recurrence. Videtic et al. [18] report on a similar case report, using radiation and cisplatin for an unresectable tracheal tumour. The patient was disease free at 2 years in this report. Although there is lack of higher-level evidence, concurrent chemoradiation may be considered as an alternative therapy for advanced tracheal adenoid cystic carcinoma.

The defining factor in our case is the deliberate decision of laryngeal preservation in managing advanced tracheal adenoid cystic carcinoma. The intraoperative finding of submucosal disease extension, histological reporting of positive margins, and perineural and lymphovascular invasion may argue an aggressive resection. However, performing a total laryngectomy with further 5 cm tracheal resection would leave our patient aphonic with mediastinal tracheostomy, limited respiratory function, and poor quality of life without any oncological gain. The published literature well describes distant metastasis in the setting of advanced AdCC being independent of local and regional control [1, 2]. It is also reported that up to 59% of patients have positive airway margins after tracheal resection, but adjuvant RT reduces local recurrence, prompting the recommendation that local disease should not be treated with a laryngectomy [8, 19, 20]. While negative margins were found to be associated with a trend towards longer survival (but no significant difference), resection and reconstruction with tumor positive margins provide a survival advantage compared to clinically defined unresectable tumors [20]. Both Haddad et al. [21] and Misiukiewicz et al. [22] report a case series highlighting the use of concurrent chemoradiation with no surgical intervention. This enabled the authors to achieve organ preservation with excellent long-term outcomes. These reported cases differ from our case, with regard to the lack tracheal involvement; however the authors do highlight the benefits of laryngeal preservation in the management of advanced airway adenoid cystic carcinoma.

## 4. Conclusions

AdCC of the trachea is a rare malignancy that is difficult to diagnose due to the nonspecific nature of the presenting symptoms [2]. The tumor is often at an advanced stage at the time of diagnosis. Surgical resection and adjuvant radiotherapy with consideration of chemotherapy are the current treatment recommendations. Chemotherapy significantly increases morbidity with marginal gains in local control. Every case must be considered based on its own merits, given the rare presentation and sobering reality of biologically positive margins. This disease remains a significant challenge as distant failure is independent of local control and there are few proven strategies of worth for disseminated disease [1, 2].
